# A Prospective Study of Arsenic Exposure, Arsenic Methylation Capacity, and Risk of Cardiovascular Disease in Bangladesh

**DOI:** 10.1289/ehp.1205797

**Published:** 2013-05-10

**Authors:** Yu Chen, Fen Wu, Mengling Liu, Faruque Parvez, Vesna Slavkovich, Mahbub Eunus, Alauddin Ahmed, Maria Argos, Tariqul Islam, Muhammad Rakibuz-Zaman, Rabiul Hasan, Golam Sarwar, Diane Levy, Joseph Graziano, Habibul Ahsan

**Affiliations:** 1 Department of Population Health, and; 2 Department of Environmental Medicine, New York University School of Medicine, New York, New York, USA; 3 Department of Environmental Health Sciences, Mailman School of Public Health, Columbia University, New York, New York, USA; 4 U-Chicago Research Bangladesh Ltd., Dhaka, Bangladesh; 5 Department of Health Studies,; 6 Department of Medicine,; 7 Department of Human Genetics, and; 8 Comprehensive Cancer Center, The University of Chicago, Chicago, Illinois, USA

**Keywords:** arsenic, arsenic methylation capacity, Bangladesh, cardiovascular disease, case–cohort study

## Abstract

Background: Few prospective studies have evaluated the influence of arsenic methylation capacity on cardiovascular disease (CVD) risk.

Objective: We evaluated the association of arsenic exposure from drinking water and arsenic methylation capacity with CVD risk.

Method: We conducted a case–cohort study of 369 incident fatal and nonfatal cases of CVD, including 211 cases of heart disease and 148 cases of stroke, and a subcohort of 1,109 subjects randomly selected from the 11,224 participants in the Health Effects of Arsenic Longitudinal Study (HEALS).

Results: The adjusted hazard ratios (aHRs) for all CVD, heart disease, and stroke in association with a 1-SD increase in baseline well-water arsenic (112 µg/L) were 1.15 (95% CI: 1.01, 1.30), 1.20 (95% CI: 1.04, 1.38), and 1.08 (95% CI: 0.90, 1.30), respectively. aHRs for the second and third tertiles of percentage urinary monomethylarsonic acid (MMA%) relative to the lowest tertile, respectively, were 1.27 (95% CI: 0.85, 1.90) and 1.55 (95% CI: 1.08, 2.23) for all CVD, and 1.65 (95% CI: 1.05, 2.60) and 1.61 (95% CI: 1.04, 2.49) for heart disease specifically. The highest versus lowest ratio of urinary dimethylarsinic acid (DMA) to MMA was associated with a significantly decreased risk of CVD (aHR = 0.54; 95% CI: 0.34, 0.85) and heart disease (aHR = 0.54; 95% CI: 0.33, 0.88). There was no significant association between arsenic metabolite indices and stroke risk. The effects of incomplete arsenic methylation capacity—indicated by higher urinary MMA% or lower urinary DMA%—with higher levels of well-water arsenic on heart disease risk were additive. There was some evidence of a synergy of incomplete methylation capacity with older age and cigarette smoking.

Conclusions: Arsenic exposure from drinking water and the incomplete methylation capacity of arsenic were adversely associated with heart disease risk.

Millions of persons worldwide, including 13 million Americans (U.S. Environmental Protection Agency 2009) and > 50 million in Bangladesh (British Geological Survey 2007), have been chronically exposed to arsenic, a group 1 human carcinogen (International Agency for Research on Cancer 2004), through contaminated drinking water. Arsenic exposure from drinking water has been associated with cardiovascular disease (CVD) (Chen CJ et al. 1996; Chen Y et al. 2011; Chiou et al. 1997; Liao et al. 2012; Tseng et al. 2003; Yuan et al. 2007). However, prospective studies assessing susceptibility to CVD due to arsenic exposure are rare.

Arsenic in drinking water is present as inorganic arsenic (iAS). Once ingested, iAs is methylated to monomethylarsonic acid (MMA) and dimethylarsinic acid (DMA). The relative distribution of urinary arsenic metabolites varies from person to person and has been interpreted to reflect arsenic methylation capacity (Hopenhayn-Rich et al. 1996; Vahter 1999). Mechanistic studies have shown that MMA^III^ is more toxic than iAs or any of the pentavalent metabolites (Petrick et al. 2000; Styblo et al. 2000). Incomplete methylation, indicated by a high percentage of urinary MMA (MMA%), has been consistently related to cancers (Chen YC et al. 2003; Pu et al. 2007; Steinmaus et al. 2006; Yu et al. 2000), and there is some evidence of stronger associations among smokers than nonsmokers (Pu et al. 2007; Steinmaus et al. 2006). However, the association between urinary MMA% and CVD risk is unknown, and research on the combined effects of arsenic and biomarkers of arsenic susceptibility on CVD risk is needed.

We conducted a prospective case–cohort study nested in a large prospective cohort to assess associations of arsenic exposure from drinking water and arsenic methylation capacity, indicated using relative distribution of urinary arsenic metabolites, with CVD risk.

## Materials and Methods

*The parent Health Effects of Arsenic Longitudinal Study (HEALS)*. Details of the study methodologies have been presented elsewhere (Ahsan et al. 2006). Briefly, between March and June 2000 (prior to recruitment), we collected water samples and geographic coordinates for 5,966 contiguous drinking-water wells in a well-defined geographic area of 25 km^2^ in Araihazar, Bangladesh (Parvez et al. 2006). Between October 2000 and May 2002, we recruited 11,746 men and women who were primary users of one of the tested wells, designated as the “index” well, for ≥ 3 years. The response rate was 97.5% (Ahsan et al. 2006). Demographic and lifestyle data were collected using a standardized questionnaire. Trained clinicians measured blood pressure with an automatic sphygmomanometer (Chen Y et al. 2007). The cohort continues to be actively followed every 2 years with in-person visits that include a physical examination and collection of urine samples. For the present study, interim health surveys were conducted every 6 months between the biennial follow-up visits. A field clinic was established exclusively for the cohort participants to receive medical diagnoses and treatments and to facilitate follow-up (Ahsan et al. 2006). Informed consent was obtained from the study participants and the study procedures were approved by the ethical committee of the Bangladesh Medical Research Council and the institutional review boards of Columbia University and the University of Chicago.

*Selection of the subcohort.* The case–cohort design has been used to analyze cohort data efficiently when most observations are censored (nondiseased) at the end of follow-up (Prentice 1986). The case–cohort design has the advantage that the subcohort can be used as controls for multiple different case groups arising from the cohort (Wacholder 1991). In addition, it preserves the ability of assessing interaction on the additive scale between exposure and potential effect modifiers such as age and sex, which are often matched in nested case–control studies.

Data on well-water arsenic exposure levels were available for all cohort participants. From the 11,224 participants who gave urine samples at baseline (95.6% of all cohort members), a 10% random sample (*n* = 1,109) was selected as the subcohort.

*Selection of the cases.* Our outcome of interest was incident fatal and nonfatal cases of CVD coded to the *International Classification of Diseases, 10th Revision* [ICD-10; World Health Organization (WHO) 2007] (ICD-10 codes I00–I99), including fatal and nonfatal stroke (codes I60–I69) and fatal and nonfatal cases of heart disease, which occurred after baseline and before 18 March 2009 (the end of the third follow-up). We *a priori* included cases of ischemic heart disease and other heart disease in a combined category of heart disease (codes I20–I25 and I30–I52) (Chen Y et al. 2011). We adapted a validated verbal autopsy procedure that was developed by the International Centre for Diarrheal Disease Research, Bangladesh (ICDDR,B), in collaboration with the WHO, to ascertain the cause of deaths in cohort participants (Chen Y et al. 2011). The ICDDR,B has used this method to ascertain causes of deaths since 1971 (Ronsmans et al. 1998) and has documented an overall 95% specificity and 50–80% sensitivity for deaths due to CVD (Alam et al. 2004). During the follow-up, upon receipt of a death report from family or neighbors, a study physician and a trained social worker administered the verbal autopsy form to the next of kin. Medical records and death certificates were collected for approximately 5% of deaths in the present study. An outcome assessment committee, consisting of physicians and a consulting cardiologist and neurologist who were blinded to exposure status, reviewed these data monthly. Causes of deaths were coded according to the WHO classification (Aho et al. 1980) and the ICD-10 (WHO 2007).

For nonfatal stroke and ischemic heart disease, potential cases and participants with heart disease symptoms including high blood pressure, chest pain, shortness of breath, irregular heartbeat, and/or palpitations were identified during our biennial follow-up visits, interim health surveys, or among individuals who came to the field clinic for medical treatment. An appointment at the field clinic was scheduled, and field staff visited the homes of participants who failed to report on the scheduled date in order to schedule a new appointment within 2 weeks. Participants were referred to three trained physicians, who were blinded to arsenic exposure information, for further evaluation and diagnostic tests at the field clinic, followed by treatment and referral to the only local hospital in the study area. All hospitalizations and records of standard diagnostic tests were requested and reviewed by the outcome assessment committee. Nonfatal stroke was defined based on WHO criteria as “rapidly developing clinical signs of focal disturbance of cerebral function, lasting > 24 hr with no apparent cause other than that of vascular origin” (Aho et al. 1980). Nonfatal ischemic heart disease was defined by meeting at least two of the three following criteria: *a*) ischemic cardiac pain determined to be typical angina, *b*) cardiac enzyme abnormalities defined as abnormal CPK-MB fraction or troponin values (three times the upper limit of normal), and *c*) specific ST-T wave and Q wave electrocardiographic abnormalities.

*Arsenic exposure measurements.* Total arsenic concentration was determined by graphite furnace atomic-absorption spectrometry (GFAAS) with a Hitachi Z-8200 (Hitachi, Tokyo, Japan) system (Cheng et al. 2005). Samples that fell below the detection limit of 5 µg/L were subsequently analyzed by inductively coupled plasma mass spectrometry (ICP-MS), with a detection limit of 0.1 µg/L (Cheng et al. 2004). Analyses of time-series samples collected from 20 tube wells monitored for 3 years in the study area showed that the arsenic concentration in well water was relatively stable over time (Cheng et al. 2005). We used the arsenic concentration in the index well assessed at baseline as the well-water arsenic exposure level. In the study population, 88% of the study participants used the index well as their exclusive source of drinking water at baseline. The average duration of index well use was 7.4 years prior to baseline, accounting for ≥ 20% of each participant’s lifetime.

Spot urine samples were collected in 50-mL acid-washed tubes from 95.6%, 94.5%, and 91.2% of the cohort participants at baseline, the first follow-up, and second follow-up, respectively. Total urinary arsenic concentration was measured by GFAAS, using a PerkinElmer A Analyst 600 graphite furnace system (PerkinElmer, Waltham, MA, USA) (Nixon et al. 1991). Urinary creatinine was analyzed using a method based on the Jaffe reaction for adjustment of urinary total arsenic concentration (Slot 1965).

*Measurement of urinary arsenic metabolites*. Urinary arsenic metabolites were analyzed using a method described by Reuter et al. (2003). This method employed high-performance liquid chromatography separation of arsenobetaine (AsB), arsenocholine (AsC), As^V^, As^III^, MMA, and DMA, followed by detection by ICP-MS using a dynamic reaction cell. The detection limits were 0.2 µg/L for AsB and AsC, and 0.1 µg/L for all other metabolites. Because As^III^ can oxidize to As^V^ during sample transport, storage, and preparation, here we report total iAs (i.e., As^III^ + As^V^). iAs and MMA were undetectable in three and seven urine samples, respectively. All the urine samples had detectable DMA. Measurements below the detection limit were assigned a value of 1/2 the detection limit. The percentage of iAs, MMA, and DMA in urine was calculated by dividing the concentration of each metabolite by the sum of the urinary concentrations of iAs, MMA, and DMA. We also constructed two methylation indices: *a*) the primary methylation index (PMI; the ratio of MMA to iAs), and *b*) the secondary methylation index (SMI; the ratio of DMA to MMA). The intraclass correlations for urinary arsenic metabolites for 3 months were all > 0.65, with the intraclass correlation estimates for MMA%, DMA%, and SMI all > 0.82.

*Statistical analyses.* We computed person-years from baseline to the date of the first CVD event or the date of the third follow-up visit. We estimated adjusted hazard ratios (aHRs) and their 95% confidence intervals (CIs) for CVD, stroke, and heart disease in relation to tertiles of baseline well-water arsenic and arsenic metabolite indices (iAs%, MMA%, DMA%, PMI, and SMI) determined in the subcohort using Cox proportional hazards models with the survival and survey packages in R, version 2.13.1 (R Foundation, Vienna, Austria). The follow-up data for the subcohort (including those who developed disease) were treated as representative of the person-time experience of the overall cohort, and the data from controls in the subcohort were weighted by 1/10, the inverse of the sampling fraction from the source population (i.e., the 11,224 participants in the full cohort who provided urine samples at baseline). The use of such weights allows the efficient use of subcohort data and data from cases not included in the subcohort (Barlow et al. 1999; Breslow et al. 2009). Standard errors were estimated using the robust variance estimator proposed by Barlow (Barlow 1994; Barlow et al. 1999). We adjusted for CVD risk factors that might be related to the influence of arsenic exposure, including sex, age (years), body mass index (BMI; kilograms per meter squared), educational attainment (years), smoking status (never/ever), hypertension (systolic blood pressure of ≥ 140 and/or diastolic blood pressure of ≥ 90 mmHg), and diabetes status (yes/no) at baseline (Chen Y et al. 2010). Age, BMI, and educational attainment were entered as continuous variables in the models. Potential confounders were selected by comparing models with and without each variable, and models with and without combinations of variables that had an impact on the association of arsenic metabolite indices or well-water arsenic with CVD risk. Subjects with missing data on BMI (*n* = 12) or hypertension (*n* = 9) were excluded, and a separate dummy variable was used for missing data on baseline diabetes status (*n* = 69) under a “missing at random” assumption. Additional adjustment for well-water arsenic or total urinary arsenic did not materially change the effect estimates (data not shown). aHRs associated with well-water arsenic were additionally adjusted for change in urinary arsenic between visits, which was associated with baseline well-water arsenic status and may be related to health effects. Natural cubic splines with two internal knots placed at the 33rd and 66th percentiles and boundary knots at the 5th and 95th percentiles were fitted to estimate nonlinear associations between urinary MMA% and heart disease (Wahba 1990; Wood 2006), with MMA% below the lower boundary knot used as the reference level of exposure. For the analyses pertaining to heart disease, sensitivity analyses were conducted excluding disease categories other than ischemic heart disease (*n* = 27). Analyses excluding participants with arsenic metabolites under the detection limit (*n* = 10) generated nearly identical results (data not shown).

We explored the combined and single effects of urinary MMA%, DMA%, and SMI with key host characteristics (sex, age, BMI, and smoking status) and well-water arsenic on heart disease risk. We assessed the presence of synergy by testing whether the combined effect of arsenic exposure and a potential effect modifier was greater than the sum of their independent effects. We estimated relative excess risk for interaction (RERI) (Rothman and Greenland 1986) and its 95% CI using the standard delta method (Hosmer and Lemeshow 1992). RERI > 0 indicates the presence of synergy of two risk factors, and a 95% CI that is positive and excludes zero corresponds to *p* < 0.05 for RERI. aHRs for combined and single effects between SMI and other variables are not reported here because they were consistent with the results for MMA% and DMA%. All analyses were conducted using R, version 2.13.1.

## Results

A total of 369 cases of CVD were identified, including 211 cases of heart disease (68 fatal and 116 nonfatal cases of ischemic heart disease, and 27 deaths from other types of heart disease); 148 cases of stroke cases (91 fatal and 57 nonfatal cases); and 10 deaths due to pulmonary heart disease, hypertensive heart disease, or multiple valve diseases. Forty case participants were also included in the 1,109-member subcohort, which was representative of the overall cohort in terms of demographic, lifestyle, and arsenic exposure variables [see Supplemental Material, Table S1 (http://dx.doi.org/10.1289/ehp.1205797)].

Cases of CVD were more likely to be men, older, and ever-smokers at baseline, compared to the subcohort (Table 1). Cases of CVD, and cases of heart disease specifically, had a higher education level and higher well-water arsenic at baseline. Cases of CVD, heart disease, and stroke were more likely to have diabetes, higher systolic blood pressure, and higher diastolic blood pressure at baseline. Compared with the subcohort members, cases had a lower average of urinary iAs% and SMI and a higher average of urinary MMA% and PMI.

**Table 1:** Baseline characteristics of subcohort members and participants with incident CVD.^*a*^

Characteristic	CVD^*b*^	Heart disease^*b*^	Stroke^*b*^	Subcohort
Participants (*n*)	369	211	148	1,109
Male (%)	74.0	70.6	77.7	42.9
Age (years)	48.5 ± 9.3	46.3 ± 9.5	51.5 ± 8.4	37.1 ± 10.1
BMI (kg/m^2^)	20.1 ± 3.7	20.7 ± 4.0	19.4 ± 3.2	19.9 ± 3.4
Education level (years)	3.9 ± 4.2	4.3 ± 4.3	3.2 ± 4.0	3.5 ± 3.8
Cigarette smoking status (%)				
Ever-smokers in men	88.6	88.6	89.6	76.9
Ever-smokers in women	19.8	24.2	12.1	6.8
Systolic blood pressure (mmHg)	130.7 ± 26.5	126.4 ± 24.2	137.6 ± 28.8	114.4 ± 17.5
Diastolic blood pressure (mmHg)	81.7 ± 14.7	80.2 ± 13.3	84.5 ± 16.3	74.1 ± 11.5
Diabetes status (%)	7.5	5.7	9.9	2.1
Well-water arsenic (µg/L)	104.2 ± 113.8	110.5 ± 123.4	95.5 ± 100.4	96.4 ± 111.7
Total urinary arsenic (µg/g creatinine)	259.9 ± 235.7	268.0 ± 247.5	249.4 ± 220.6	277.1 ± 356.3
Urinary iAs%	14.3 ± 6.1	14.5 ± 6.1	14.1 ± 6.2	15.9 ± 7.1
Urinary MMA%	14.4 ± 5.3	14.5 ± 5.4	14.3 ± 5.3	12.8 ± 5.1
Urinary DMA%	71.3 ± 8.1	71.0 ± 8.6	71.7 ± 7.6	71.3 ± 8.9
PMI [MMA/(As^III^+As^V^)]	1.2 ± 0.6	1.2 ± 0.6	1.2 ± 0.7	1.0 ± 0.8
SMI (DMA/MMA)	5.8 ± 2.9	5.8 ± 3.1	5.8 ± 2.7	6.7 ± 3.6
Abbreviations: PMI, primary methyla­tion index; SMI, secondary methyla­tion index. Values are mean ± SD except where indicated.^***a***^Data on BMI, systolic blood pressure, diastolic blood pressure, and diabetes status were missing for 12, 9, 9, and 105 participants, respectively. ^***b***^CVD cases include incident fatal and nonfatal cases of CVD (ICD-10 codes I00–I99); cases of heart disease include fatal and nonfatal cases of ischemic heart disease and deaths from other heart disease (codes I20–I25 and I30–I52); stroke cases include fatal and nonfatal stroke (I60–I69); 10 deaths due to pulmonary heart disease, hyper­tensive heart disease, or multiple valve diseases were not classified as heart disease or stroke cases.				

We observed an increased risk of overall CVD and heart disease in participants with higher levels of baseline well-water arsenic. Participants exposed to ≥ 108 µg/L (mean, 222.3 µg/L) of well-water arsenic were 1.49 (95% CI: 1.06, 2.11) times and 1.54 (95% CI: 1.02, 2.31) times more likely to develop CVD and heart disease, respectively, compared with their counterparts who were exposed to ≤ 25 µg/L (Table 2). The aHR for heart disease in association with a 1-SD increase in well-water arsenic (112 µg/L) was 1.20 (95% CI: 1.04, 1.38). There was no significant association between well-water arsenic and stroke risk, although the aHR associated with the highest level of well-water arsenic was elevated (aHR = 1.38; 95% CI: 0.84, 2.27).

**Table 2:** Association between baseline well-water arsenic (μg/L) and CVD risk.

Well-water arsenic (μg/L)	Mean^*a*^	Subcohort (*n*)	CVD		Heart disease		Stroke	
			Cases (*n*)	aHR (95%CI)^*b*^	Cases (*n*)	aHR (95%CI)^*b*^	Cases (*n*)	aHR (95%CI)^*b*^
0.1–25	7.2	365	114	1.00	61	1.00	50	1.00
25.1–107	59.9	364	120	1.00 (0.67, 1.50)	72	1.18 (0.75, 1.84)	46	0.86 (0.49, 1.51)
108–864	222.8	364	132	1.49 (1.06, 2.11)	75	1.54 (1.02, 2.31)	52	1.38 (0.84, 2.27)
Per 1 SD (112μg/L) increase		1,093	366	1.15 (1.01, 1.30)	208	1.20 (1.04, 1.38)	148	1.08 (0.90, 1.30)
^***a***^Category-specific mean values of well-water arsenic in the subcohort. ^***b***^Adjusted for sex, baseline age, BMI, smoking status (never and ever), educational attainment, hypertension, diabetes status, and change in urinary arsenic between visits.								

A positive association was observed between urinary MMA% and CVD risk, with an aHR of 1.55 (95% CI: 1.08, 2.23) for the top tertile (Table 3). The aHRs for heart disease in increasing MMA% tertiles were 1.00 (reference), 1.65 (95% CI: 1.05, 2.60), and 1.61 (95% CI: 1.04, 2.49); however, the effect estimates did not suggest a linear dose–response relationship between urinary MMA% above the first tertile and risk of heart disease. Participants with moderate DMA% (68.7–75.5%) had a significantly reduced heart disease risk. A similar but nonsignificant association was observed between high DMA% and heart disease risk. Participants with an SMI ≥ 7.2 had a statistically significant reduction in risk of CVD and heart disease (aHR = 0.54; 95% CI: 0.34, 0.85 and aHR = 0.54; 95% CI: 0.33, 0.88, respectively), compared with those who had an SMI ≤ 4.8. On the other hand, there was no significant association of iAs% or PMI with CVD or heart disease. There was no evidence that iAs%, MMA%, DMA%, PMI, or SMI were related to stroke risk, with the possible exception of a nonsignificant negative association between stroke and SMI ≥ 7.2 (aHR = 0.58; 95% CI: 0.31, 1.08). Well-water arsenic and total urinary arsenic were weakly correlated with MMA%, with a Spearman rank correlation of 0.10 and 0.07, respectively. The associations for ischemic heart disease only (*n* = 184 after excluding 27 deaths due to nonischemic heart disease) were similar to those for all heart disease cases. For instance, the aHRs for increasing MMA% tertiles were 1.00 (reference), 1.68 (95% CI: 1.04, 2.72), and 1.64 (95% CI: 1.03, 2.59).

**Table 3:** Associations between urinary arsenic metabolite indices and CVD risk.

Urinary arsenic metabolicindex	Mean^*a*^	Subcohort (*n*)	CVD		Heart disease		Stroke	
			Cases (*n*)	aHR (95%CI)^*b*^	Cases (*n*)	aHR (95%CI)^*b*^	Cases (*n*)	aHR (95%CI)^*b*^
iAs%^*c*^
0.3–12.4	9.3	363	157	1.00	92	1.00	63	1.00
12.5–17.3	14.8	367	118	1.28 (0.90, 1.81)	60	1.12 (0.74, 1.68)	53	1.39 (0.84, 2.29)
17.4–69.3	23.2	363	91	1.05 (0.71, 1.56)	56	1.17 (0.76, 1.80)	32	0.87 (0.49, 1.57)
MMA%^*c*^
0.2–10.3	7.7	366	74	1.00	40	1.00	32	1.00
10.4–14.3	12.3	363	131	1.27 (0.85, 1.90)	81	1.65 (1.05, 2.60)	49	0.91 (0.51, 1.61)
14.4–33.8	18.6	364	161	1.55 (1.08, 2.23)	87	1.61 (1.04, 2.49)	67	1.35 (0.81, 2.27)
DMA%^*c*^
27.9–68.6	61.6	363	117	1.00	74	1.00	39	1.00
68.7–75.5	72.1	367	133	0.98 (0.69, 1.39)	65	0.65 (0.43, 0.98)	64	1.53 (0.91, 2.55)
75.6–99.2	80.2	363	116	0.75 (0.49, 1.14)	69	0.68 (0.44, 1.05)	45	0.90 (0.48, 1.67)
PMI^*c*^
0.01–0.66	0.48	362	83	1.00	48	1.00	34	1.00
0.67–1.05	0.85	365	98	0.93 (0.65, 1.34)	56	0.91 (0.59, 1.39)	37	0.88 (0.52, 1.51)
1.06–19.57	1.61	363	185	0.88 (0.61, 1.26)	104	0.91 (0.59, 1.40)	77	0.81 (0.49, 1.34)
SMI^*c*^
1.4–4.8	3.6	361	152	1.00	88	1.00	59	1.00
4.9–7.1	5.9	363	136	1.00 (0.72, 1.38)	77	1.02 (0.70, 1.48)	56	1.00 (0.61, 1.64)
7.2–32.3	10.5	363	77	0.54 (0.34, 0.85)	43	0.54 (0.33, 0.88)	32	0.58 (0.31, 1.08)
Abbreviations: PMI, primary methyla­tion index; SMI, secondary methyla­tion index.^***a***^Category-specific mean values of urinary arsenic metabolites in the subcohort. ^***b***^Adjusted for sex, baseline age (years), BMI, smoking status (never and ever), educational attainment, hypertension, and diabetes status. ^***c***^Cut points were determined by tertiles in the subcohort.								

**Figure 1:**
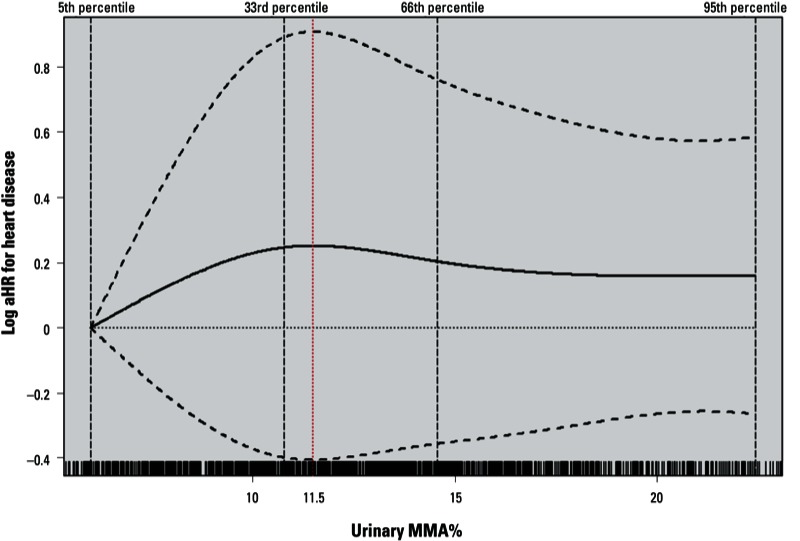
Log aHRs for incident heart disease according to urinary MMA% modeled as a natural cubic spline with internal knots placed at the 33rd and 66th percentiles and boundary knots at the 5th and 95th percentiles adjusted for sex, baseline age (years), BMI, smoking status (never and ever), educational attainment, hypertension, and diabetes status. The ticks at the bottom represent urinary MMA% values and the dashed black vertical lines represent the location of knots used for fitting the cubic spline. The dashed curved lines indicate the 95% CIs of the log aHRs.

We further explored the nonlinear association between urinary MMA% and heart disease risk. The data suggested a nonlinear association (Figure 1), such that the log aHRs for heart disease increased with increasing urinary MMA% through the first third of the distribution, but leveled off for higher levels of MMA%.

**Figure 2:**
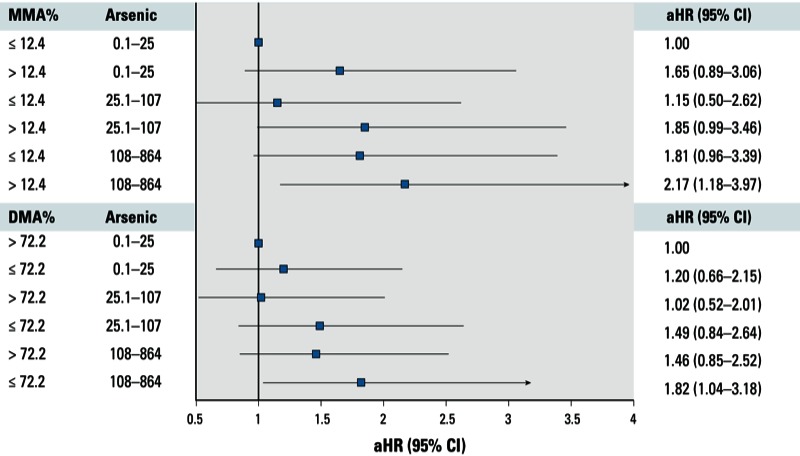
Estimated combined effects (aHRs and 95% CIs) of urinary MMA% and DMA% with well-water arsenic on heart disease risk, adjusted for sex, baseline age (years), BMI, smoking status (never and ever), educational attainment, hypertension, diabetes status, and change in urinary arsenic between visits.

Compared with younger persons with lower MMA%, heart disease risk among those who were older and had higher MMA% (aHR = 3.88; 95% CI: 2.12, 7.09) was greater than that among those who were older and had a lower MMA% (aHR = 2.85; 95% CI: 1.53, 5.31) (Table 4). Compared with never-smokers with lower MMA%, heart disease risk among ever-smokers who had higher MMA% (aHR = 3.82; 95% CI: 2.04, 7.14) was greater than that among ever-smokers who had lower MMA% (aHR = 2.58; 95% CI: 1.30, 5.11). The pattern of the associations was similar when DMA% was considered. The combined effects of higher MMA% and lower DMA% with older age and ever-smoking were greater than the sum of the individual effects (all RERI > 0), indicating synergistic effects; however, the estimates were not precise and not statistically significant. There was no apparent synergistic effect of sex and BMI with higher MMA% or lower DMA% [see Supplemental Material, Table S2 (http://dx.doi.org/10.1289/ehp.1205797)]. At each level of well-water arsenic, heart disease risk was higher among those with higher MMA% or lower DMA% (Figure 2). Compared with participants with the lowest level of well-water arsenic and lower MMA% (≤ 12%), those with moderate (25.1–107 µg/L) or high levels of well-water arsenic (108–864 µg/L) were more likely to develop heart disease, and participants exposed to high levels of arsenic and also had higher MMA% or lower DMA% were most likely to develop heart disease (aHR = 2.17 and 1.82, respectively). The RERI for synergistic effect between well-water arsenic and higher MMA% was 0.05 (95%CI: –1.25, 1.36) at moderate levels of well-water arsenic and was –0.29 (95% CI: –1.73, 1.14) at high levels of well-water arsenic. The RERI for synergistic effect between well-water arsenic and lower DMA% was 0.28 (95% CI: –0.76, 1.31) and 0.16 (95% CI: –0.95, 1.26) at moderate and high levels of well-water arsenic, respectively.

**Table 4:** Estimated combined effects of urinary metabolite indices and baseline age and smoking status on heart disease risk.

Urinary arsenic metabolite index	Combined effect between urinary arsenic metabolite indices and age				Combined effect between urinary arsenic metabolite indices and smoking			
	Age^*a*^	Cases/subcohort (*n*)	aHR (95%CI)^*b*^	RERI (95%CI)	Smoking status	Cases/subcohort (*n*)	aHR (95%CI)^*c*^	RERI (95%CI)
MMA%^*a*^
≤12.4	≤36	15/305	1.00		Never	35/392	1.00	
> 12.4	≤36	13/255	0.94 (0.43, 2.01)		Never	28/295	1.29 (0.75, 2.20)	
≤12.4	>36	61/244	2.85 (1.53, 5.31)		Ever	41/157	2.58 (1.30, 5.11)	
> 12.4	>36	119/289	3.88 (2.12, 7.09)	1.09 (–0.28, 2.47)	Ever	104/249	3.82 (2.04, 7.14)	0.96 (–0.63, 2.55)
DMA%^*a*^
>72.2	≤36	11/282	1.00		Never	38/374	1.00	
≤72.2	≤36	17/278	1.41 (0.64, 3.07)		Never	25/313	1.25 (0.73, 2.15)	
>72.2	>36	96/263	3.67 (1.81, 7.43)		Ever	69/171	2.81 (1.56, 5.05)
≤72.2	>36	84/270	4.99 (2.53, 9.83)	0.92 (–0.83, 2.66)	Ever	76/235	3.93 (2.14, 7.22)	0.88 (–0.81, 2.56)
^***a***^Cut points were determined by median values in the subcohort. ^***b***^Adjusted for sex, baseline BMI, smoking status (never and ever), educational attainment, hypertension, and diabetes status. ^***c***^Adjusted for sex, baseline age (years), BMI, educational attainment, hypertension, and diabetes status.								

## Discussion

In this prospective study, we found an increased risk of fatal and nonfatal CVD, especially heart disease, associated with higher well-water arsenic concentration and lower methylation capacity, indicated by higher urinary MMA% or lower DMA%. We observed that the association between urinary MMA% and heart disease was nonlinear, such that the association increased with increasing MMA% at lower levels, and then appeared to level off. The effects associated with higher levels of well-water arsenic and lower methylation capacity on heart disease risk were at least additive. There was some evidence of a small synergistic effect between lower methylation capacity and older age or cigarette smoking, although the estimates were imprecise.

We have previously reported a significant positive association between arsenic exposure at moderate-to-high levels (> 100 µg/L) and CVD mortality, especially heart disease mortality in the population, with 198 CVD deaths (Chen Y et al. 2011). The present study adds to the evidence supporting a causal relation between arsenic exposure and heart disease with findings supported by a much larger sample size with 369 fatal and nonfatal CVD cases. In our data, the association between arsenic exposure and stroke risk was not significant. This finding was in line with two previous ecological studies of high levels (> 300 µg/L) of arsenic exposure (Wu et al. 1989; Yuan et al. 2007) but not with a cross-sectional study of moderate levels (median < 140 µg/L in various villages) (Chiou et al. 1997). Importantly, in the present study, we also found a positive association between urinary MMA%—a biomarker specific for susceptibility to the health effects of arsenic exposure—and heart disease risk, and the association between MMA% and stroke risk was weaker and nonsignificant, suggesting the effect of arsenic exposure on heart disease risk was stronger than that on stroke risk. However, stroke is a heterogeneous disease that comprises subtypes with different etiologies, and future studies that estimate associations for subtypes of stroke are needed. It should be noted that our study is mostly informative and supportive of cardiovascular effects at moderate-to-high levels of arsenic exposure (> 100 µg/L). Findings from studies of lower levels of arsenic exposure (< 100 µg/L) from drinking water have been inconsistent (Engel and Smith 1994; Medrano et al. 2010; Meliker et al. 2007), possibly because of limitations such as narrow exposure ranges and measurement errors.

Previous studies have reported evidence of adverse effects of incomplete arsenic methylation capacity on cancer risk. The literature on the role of arsenic methylation capacity in the risk of stroke and heart disease is limited. Tseng et al. (2005) reported that among individuals with high arsenic exposure, the prevalence of peripheral vascular disease was greater among those with higher levels of MMA%. In studies of hypertension and carotid atherosclerosis from the same population, there was an insignificant positive association of urinary MMA% with the prevalence of hypertension and carotid atherosclerosis (Huang YK et al. 2007; Huang YL et al. 2009). In the present study, we also observed a significant inverse association between SMI (the ratio of DMA to MMA) and the risk of CVD and heart disease. There was no evidence that iAs% or PMI was related to CVD risk. These data suggest a more critical role of the second methylation step, or of complete methylation capacity, than the first step in arsenic methylation. In addition, compared with participants with the lowest level of well-water arsenic and higher methylation capacity, those exposed to moderate or high levels of well-water arsenic and who also had lower methylation capacity were most likely to develop heart disease (Figure 2). The data suggest a potential synergy between arsenic exposure and lower methylation capacity at moderate but not high levels of arsenic exposure. However, the estimates were imprecise. Collectively, data from the present study and previous studies suggest that individuals with suboptimal or incomplete arsenic methylation capacity are more susceptible to adverse effects of arsenic exposure, including effects on both cancer and CVD. Future studies are needed to assess whether the susceptibility due to lower methylation capacity was more critical at lower levels of exposure.

We found that the association between MMA% and heart disease risk was slightly stronger in ever-smokers compared with never-smokers, although the differences were not significant. This finding suggests that arsenic methylation capacity is likely to explain partly the synergy between arsenic exposure and smoking in heart disease risk, as observed in our previous cohort analyses on CVD mortality (Chen Y et al. 2011). Several studies have documented a synergistic effect of MMA% and smoking in the risk of bladder cancer (Steinmaus et al. 2006) and urothelial carcinoma (Pu et al. 2007). Taken together, the body of literature suggests that cigarette smoking may be an important factor that influences the health effects of arsenic exposure and arsenic methylation capacity.

The mechanisms by which arsenic leads to heart disease still remain to be elucidated. Several animal studies have suggested that arsenic can induce atherosclerosis (Cheng et al. 2011; Lemaire et al. 2011; Simeonova et al. 2003; Srivastava et al. 2009) and high blood pressure (Sanchez-Soria et al. 2012), possibly by induction of oxidative stress, inflammatory responses, and endothelial dysfunction as reviewed by Navas-Acien et al. (2005). Epidemiologic studies have suggested that arsenic may increase heart disease risk through its effects on subclinical cardiovascular outcomes, as evidenced by the positive associations of arsenic exposure with the prevalence of QTc prolongation (Chen Y et al. 2013b; Mordukhovich et al. 2009; Mumford et al. 2007) and subclinical anthrosclerosis (Chen Y et al. 2013a; Wang et al. 2002). Future studies on effects of arsenic on preclinical phenotypes relevant for heart disease are needed to clarify the underlying mechanisms.

Several aspects should be taken into consideration when interpreting the results of our study:

Although participants had on average used the baseline wells for 7.4 years, we did not have a complete history of lifetime exposure, and some participants did not use the baseline well exclusively. Changes in exposure level since baseline also occurred in some participants (Chen Y et al. 2007). The potential misclassification of exposure is probably unlikely to be differential by subsequent disease status. However we could not estimate the extent of the potential measurement errors.
We were unable to distinguish and quantify MMA^III^ and MMA^V^ separately in urine. MMA^III^ is very unstable and must be measured or stabilized immediately after collection, making it impractical for this study design (Valenzuela et al. 2005).
Urinary arsenic metabolites were measured in one spot urine sample. The literature suggests that arsenic methylation efficiency of an individual is stable over time (Concha et al. 2002; Navas-Acien et al. 2009). However, we were not able to estimate the effects of arsenic metabolites at different time periods or during specific time windows, nor could we estimate the latency of associations.
CVD deaths were ascertained using the verbal autopsy procedure, which may involve some misclassification. We might also have missed some nonfatal cases because the ascertainment relies partly on participant visits to the field clinic. However, there was no evidence that factors related to access to health care, such as socioeconomic variables including occupation, education, and land ownership were associated with arsenic exposure levels in the cohort (Chen Y et al. 2010). Although we believe that potential misclassification of CVD deaths and incomplete ascertainment of nonfatal cases was unlikely to have been differential by arsenic levels, we cannot predict the exact direction or extent of the potential bias with certainty.
Finally, although we did not collect information on lipid profiles at baseline, available literature does not suggest a positive association between arsenic exposure and lipid profiles, nor was there evidence that the association between arsenic exposure and CVD is modifiable by lipid profiles (Simeonova et al. 2003; Tseng et al. 1997).

## Conclusions

We observed a positive association of arsenic exposure from drinking water and the proportion of MMA in urine with the risk of CVD, especially heart disease. The risk of heart disease associated with higher levels of arsenic exposure and incomplete methylation capacity, indicated by higher urinary MMA% or lower urinary DMA%, was greater than the risk associated with the same levels of arsenic but better methylation capacity. The data also suggest a possible synergy between incomplete methylation and older age, and between incomplete methylation capacity and cigarette smoking, although the estimates were not precise. These findings stress that arsenic methylation capacity is a susceptible factor for the cardiovascular effects of arsenic exposure.

## Supplemental Material

(561 KB) PDF
