# Supplementary material for: A Prospective Study of Arsenic Exposure, Arsenic Methylation Capacity, and Risk of Cardiovascular Disease in Bangladesh
Source: Environ Health Perspect. 2013 May 10;121(7):832–8. doi: 10.1289/ehp.1205797 (PMC3701993; doi:10.1289/ehp.1205797)
Supplement: (561 KB) PDF [file ehp.1205797.s001.pdf]

Supplemental Material

A Prospective Study of Arsenic Exposure, Arsenic Methylation Capacity, and Risk of Cardiovascular Disease in Bangladesh

Yu Chen, Fen Wu, Mengling Liu, Faruque Parvez, Vesna Slavkovich, Mahbub Eunus, Alauddin Ahmed, Stephanie Segers, Maria Argos, Tariqul Islam, Muhammad Rakibuz-Zaman, Rabiul Hasan, Golam Sarwar, Diane Levy, Joseph Graziano, and Habibul Ahsan

Table of Contents:

Table S1. Characteristics of subcohort members in the present study (*n* = 1,109) and participants in the overall cohort study (*n* = 11,746) [*n* (%) or mean ± SD]……………………………….2

Table S2. Estimated joint effects of sex and baseline BMI, and urinary metabolite indices on heart disease risk……………………………………………………………………………3

Table S1. Characteristics of subcohort members in the present study (n = 1,109) and participants in the overall cohort study (n = 11,746) [n (%) or mean ± SD].

	Subcohort members	Overall cohort participants
Sex		
Women	633 (57.1)	6704 (57.1)
Men	476 (42.9)	5042 (42.9)
Age (years)	37.1 ± 10.1	37.1 ± 10.1
Body mass index (kg/m^2)	19.9 ± 3.4	19.8 ± 3.2
Education (years)	3.5 ± 3.8	3.5 ± 3.8
Smoking status		
Never	700 (63.1)	7568 (64.5)
Past	59 (5.3)	777 (6.6)
Current	350 (31.6)	3395 (28.9)
Systolic blood pressure (mmHg)	114.4 ± 17.5	114.7 ± 17.9
Diastolic blood pressure (mmHg)	74.1 ± 11.6	74.0 ± 11.8
Well arsenic (µg/L)	96.4 ± 111.7	101.5 ± 115.4
Urinary arsenic (µg/L)	136.1 ± 155.6	122.7 ± 136.4
Urinary creatinine (mg/dL)	59.7 ± 46.5	62.1 ± 45.4
Urinary arsenic (µg/g creatinine)	277.1 ± 356.2	233.0 ± 233.1

Table S2. Estimated joint effects of sex and baseline BMI, and urinary metabolite indices on heart disease risk.

Urinary arsenic metabolite indices	Joint effect between urinary arsenic metabolite indices and sex				Joint effect between urinary arsenic metabolite indices and BMI			
	Sex	Cases/subcohort, *n*	HR (95% CI)[a]	RERI (95% CI)	BMI[b]	Cases/subcohort, *n*	HR (95% CI)[c]	RERI (95% CI)
MMA%[b]								
≤ 12.4	Men	42/159	1.00		≤ 19.3	27/261	1.00	
>12.4	Men	106/314	1.43 (0.88, 2.33)		≤ 19.3	61/284	1.62 (0.98, 2.65)	
≤ 12.4	Women	34/390	1.37 (0.70, 2.65)		> 19.3	49/288	1.37 (0.76, 2.49)	
>12.4	Women	26/230	1.89 (0.95, 3.76)	0.09 (-0.99, 1.17)	> 19.3	71/260	1.74 (1.03, 2.95)	-0.24 (-1.27, 0.78)
DMA%[b]								
> 72.2	Men	69/191	1.00		≤ 19.3	38/250	1.00	
≤ 72.2	Men	79/282	1.45 (0.93, 2.26)		≤ 19.3	50/295	1.43 (0.88, 2.32)	
> 72.2	Women	38/354	1.48 (0.81, 2.70)		> 19.3	69/295	1.21 (0.72, 2.03)	
≤ 72.2	Women	22/266	1.71 (0.84, 3.45)	-0.22 (-1.30, 0.86)	> 19.3	51/253	1.55 (0.95, 2.52)	-0.09 (-0.97, 0.80)

Abbreviations: BMI, body mass index; HR, hazard ratio; CI, confidence interval; RERI: relative excess risk for interaction; MMA, monomethylarsonic acid; DMA, dimethylarsinic acid; SMI, secondary methylation index.

[a]Adjusted for baseline age (years), body mass index, smoking status (never and ever), educational attainment, hypertension, and diabetes status.

[b]Cut points were determined by median values in the subcohort.

[c]Adjusted for sex, baseline age (years), smoking status (never and ever), educational attainment, hypertension, and diabetes status.
